# Gene editing for Spinocerebellar ataxia type 3 taking advantage of the human *ATXN3L* paralog as replacement gene

**DOI:** 10.1038/s41434-025-00557-2

**Published:** 2025-07-28

**Authors:** Margareta Rybarikova, Maria Rey, Ed Hasanovic, Mélanie Sipion, Lukas Rambousek, Nicole Déglon

**Affiliations:** 1 Lausanne University Hospital (CHUV) and University of Lausanne (UNIL), Department of Clinical Neurosciences (DNC), Laboratory of Cellular and Molecular Neurotherapies, Lausanne, Switzerland; 2 Lausanne University Hospital (CHUV) and University of Lausanne (UNIL), Neuroscience Research Center (CRN), Laboratory of Cellular and Molecular Neurotherapies (LCMN), Lausanne, Switzerland; 3 Lausanne University Hospital (CHUV) and University of Lausanne (UNIL), Neurorestore, Lausanne Switzerland

**Keywords:** Cell death in the nervous system, Molecular biology, Cellular neuroscience

## Abstract

Spinocerebellar ataxia type 3 (SCA3) is a rare neurodegenerative disease caused by a CAG expansion of the ataxin-3 gene (*ATXN3*). SCA3 patients suffer from ataxia, spasticity and dystonia in mid-adulthood, with spinocerebellar dysfunction and degeneration. As a monogenic disease for which only symptomatic treatment is available, *ATXN3* is an attractive target for gene editing. We used the KamiCas9, a self-inactivating gene editing system, to explore gene editing strategies suitable for all SCA3 patients. We first tested the deletion of exon 10 or the introduction of a premature stop codon into exon 9. High editing events were observed in vitro, but efficiency was very low in SCA3 transgenic mice. We then evaluated an ablate-and-replace strategy. The ablate experiments resulted in 55 ± 18% cerebellar editing of the *ATXN3* gene. A human *ATXN3L* paralog, expressed in the brains of SCA3 patients, may act as a natural, CRISPR-resistant replacement gene. In a proof-of-principle study, ablate and ablate-and-replace strategies were evaluated in SCA3 transgenic mice. Two months after injection, similar editing efficiencies were obtained in the ablate and ablate-and-replace groups. Immunofluorescence and RT-qPCR analyses of cerebellar markers support the development of this strategy for SCA3 treatment.

## Introduction

Spinocerebellar ataxia type-3 (SCA3) or Machado-Joseph disease (MJD) [1] (MIM#109150) is the most common type of spinocerebellar ataxia (SCA) worldwide, with a prevalence of 1–5 per 100,000. There are 13–42 CAG repeats in exon 10 of the ataxin-3 (*ATXN3*) gene in healthy individuals, and 60–84 repeats in individuals with SCA3 [2]. SCA3 is characterized by progressive degeneration in specific brain regions, including the deep cerebellar nuclei (DCN), brainstem nuclei, spinocerebellar tracts, thalamus and globus pallidus [3].

The *ATXN3* gene encodes a 361-amino acid protein with a molecular weight of ~42 kDa (P54252-2, Uniprot). The N-terminal region contains the enzymatically active Josephin domain, whereas the C-terminal region features three ubiquitin-interaction motifs (UIMs) and the polyglutamine (polyQ) tract. The physiological function of the ATXN3 protein remains to be fully elucidated, but it has been shown to act as a deubiquitinating enzyme (DUB) in the ubiquitin-proteasome pathway [4]. ATXN3 does not appear to be essential for the brain, as demonstrated by the absence of a phenotype in knockout mice [4].

SCA3 is an autosomal dominant disorder. The strategies developed in recent years have therefore focused on lowering levels of *ATXN3* expression, with the aim of partially decreasing the levels of mutant and/or wild-type ATXN3. These strategies are based on RNA interference (RNAi) or antisense oligonucleotides (ASO) [5–8]. We have shown that targeting a single nucleotide polymorphism (SNP) associated with the expanded CAG repeat that is present in ~70% of patients efficiently silences the mutant *ATXN3* [9]. The knockdown of endogenous wild-type ATXN3 in the adult rat brain was well tolerated, supporting the hypothesis that ATXN3 has redundant functions [10]. The emergence of gene-editing platforms [11] has further expanded the possibilities for preventing *ATXN3* neurotoxicity. In particular, strategies targeting the *ATXN3* gene enable one-time treatment with the potential for permanent therapeutic benefits. However, all published studies to date have been limited to in vitro models of SCA3 [12, 13].

In this study, we made use of several unique features of *ATXN3* to evaluate in vivo gene editing strategies. Firstly, there are splice isoforms of the *ATXN3* gene lacking the third UIM and CAG repeats but with similar levels of enzymatic activity [14]. Secondly, there is an intron-free paralog present in humans — *ATXN3L*, 85% sequence identity — which is thought to have resulted from a retrotransposition event in primates [15–17]. The CAG repeats do not appear to expand because of GAA interruptions [18]. It is therefore possible to inactivate (ablate) the human mutant and wild-type *ATXN3* and rely on *ATXN3L* as a CRISPR-resistant replacement gene. Gene replacement was initially developed for loss-of-function genetic disorders, with the aim of replacing the non-functional gene [19, 20]. For autosomal dominant diseases, including polyglutamine disorders, current therapeutic strategies focus principally on allele-specific inactivation/silencing, but clinical development is complex and not all patients are eligible for such treatment [21, 22]. An ablate-and-replace approach would provide an attractive alternative that is easier to implement and suitable for use in all patients [23, 24].

## Materials and methods

### Human embryonic kidney 293 T (HEK293T) cells

HEK293T cells (mycoplasma-negative, ATCC, LGC Standards GmbH, Wessel, Germany) were cultured in DMEM-Glutamax supplemented with 10% FBS and 1% penicillin/streptomycin (Gibco, Life Technologies, Zug, Switzerland) at 37 °C under an atmosphere containing 5% CO_2_. Cells were passaged twice weekly after trypsin treatment for dissociation (Gibco, Life Technologies, Zug, Switzerland) and plated at a density of 2 × 10^6^ cells/cm^2^ in T175 flasks.

### Neuronal progenitor cells culture and transduction

The protocol for culturing human neural progenitor cells (NPCs) was described previously [25]. We plated 6 × 10^6^ cells per 6-well plates and one day later, cells were trypsinized and splited in two 6-wells. The day after, the NPCs were transduced with LV (SIN-cPPT-PGK-SpCas9-BPNLS-WPRE [25]; SIN-cPPT-U6-sgATXN2-PGK-mCherry-WPRE, ratio1:3 120 ng/p24) diluted in N2B27 medium (5% of the culture volume) with 10 ng/ml EGF, 10 ng/ml FGF and 20 ng/ml BDNF). The medium was replaced 6 and 72 h after transduction. The cells were lysed for DNA and protein extraction, 6 days post-transduction.

### Plasmids

A detailed description of all plasmids is provided in the supplemental Materials and Methods section.

### Transfection of HEK293T cells

HEK293T cells have 14 (3QK-10Q) and 21 CAG repeats (3QK-17Q) in both alleles of ATXN3 [12]. For evaluation of the editing efficiencies of the various sgRNAs, HEK293T cells were plated at a density of 5 × 10^5^ cells per well in six-well plates the day before transfection. The cells were transfected at a molar ratio of 1:3 with plasmids encoding SpCas9 and sgATXN. The sgRNA was expressed under the control of the U6 promoter and all sgRNAs had a backbone containing an optimized tracrRNA, as previously described [26]. Cells transfected with the plasmid encoding the SpCas9 alone were used as a negative control.

### Production of adeno-associated vectors (AAVs) and Lentiviral vectors (LV)

AAVs were produced in HEK293T cells by calcium phosphate-mediated transfection, as previously reported [26]. The viral genome content (vg/mL) of each AAV was assessed by *Taq*Man qPCR with primers recognizing the inverted terminal repeats of the AAV2 viral genome (forward primer: GGAACCCCTAGTGATGGAGTT, reverse primer: CGGCCTCAGTGAGCGA, *Taq*Man probe: FAM-CACTCCCTCTCTGCGCGCTCG-TAMRA) and the KAPA probe fast qPCR universal kit (Sigma-Aldrich, Buchs, Switzerland). AAV vectors were stored at −80 °C until use.

LV pseudotyped with the vesicular stomatitis virus glycoprotein envelope (VSV-G) were produced in HEK293T cells with as four-plasmid system as previously described [27]. The viral particle content was measured by a p24 antigen enzyme-linked immunosorbent assay (Retrotek, Kampenhout, Belgium). Viral stocks were stored at −80 °C until use.

### Animals

Wildtype C57BL/6 and transgenic and MJD84.2 [28] mice expressing the mutant human ataxin-3 gene with 84 CAG repeats were obtained from Jackson Laboratories (Bar Harbor, Maine, USA). Adult male and female WT (C57BL/6) or MJD84.2 mice (9–17 weeks old) were used for the in vivo experiments. Mice were housed in a specific pathogen-free (SPF) facility in GM500 IVC cages (Techniplast, Gams, Switzerland) or rat R.BTM.Ux/R.ICV.6 cages (Innovive, Paris, France) containing corn cob bedding and placed in Innorack single-sided rat racks (cat# RS.5.8.40), with no more than five mice per cage. The animals were maintained in a controlled-temperature room (22 ± 1 °C), under a 14-h light/10-h dark cycle. The following enrichments were provided: two pieces of wipes, one cardboard tunnel, and one cardboard or polysulfide house with two entrances/exits. Food (SAFE® 150, Safe, Rosenberg, Germany) and water were provided *ad libitum*. All experimental procedures were performed in strict accordance with Swiss regulations concerning the care and use of laboratory animals, under veterinary/ethical authorization #3682.

### Stereotaxic injections

Anesthesia and surgical procedures were performed as previously described [29]. We injected 5 μL AAV2/rh.10 per site bilaterally into the mouse cerebellum at a rate of 0.5 μL/min at the following coordinates: site 1: −5.75; ±1.6; −2.6 (−5.75 mm rostral to Bregma; ±1.6 mm lateral to midline; and −2.6 mm ventral to the skull surface, with the tooth bar set at –3.3 mm) and site 2: −6.36; ±1.6; −2.6. The needles were left in place for 5 min after injection and then slowly removed. During surgery, body temperature was controlled with a warming blanket (CMA 450 Temperature Controller, Phymep, Paris, France) and the eyes were protected with 0.2% Viscotears liquid gel (Novartis, Basel, Switzerland). Post-surgery analgesic treatment (acetaminophen, Dafalgan Upsa 1000 mg/750 mL) was administered in the drinking water for 72 h. A detailed description of all experiments is provided in the supplemental Materials and Methods section.

### Histological processing

The brains of the animals were removed, post-fixed by incubation in 4% PFA for 24 h and cryoprotected by two consecutive incubations in 20% and 30% sucrose (Sigma-Aldrich, Buchs, Switzerland) for 12 h each. A cryostat (CM1850, Leica Biosystems, Muttenz, Switzerland) with a freezing stage at −20 °C (SM2400; Leica Microsystems AG, Glattbrugg, Switzerland) was used to cut 30-μm-thick coronal brain sections. Sections were collected and stored in anti-freeze solution (0.2 M sodium phosphate buffer, 25% glycerol, 30% ethylene glycol) in 96-well plates at −20 °C until use. The brain sections were mounted directly on slides, in Vectashield supplemented with DAPI (Reactolab, Servion, Switzerland).

Brain sections for immunofluorescence staining were rinsed in TBS-T (0.9% NaCl, 0.1% Triton X-100 and 10 mM Tris pH7.6; 3 rinses, each for 10 min) at room temperature and blocked by incubation for 1 hour in TBS-T supplemented with 5% BSA (Sigma-Aldrich, Buchs, Switzerland). The following primary antibodies were used for immunofluorescence staining: a mouse monoclonal anti-glial fibrillary acidic protein (GFAP)-Cy3™ (Sigma, Buchs, Switzerland; RRID: AB_476889) diluted 1/800 in PBS-T supplemented 10% NGS, a rabbit anti-spinocerebellar ataxin type 3 antibody (Abcam, Amsterdam, Netherland; RRID: AB_2040952) diluted 1/500 in TBS-T supplemented with 1% BSA, and a polyclonal rabbit anti-calbindin antibody (Sigma, Buchs, Switzerland; RRID: AB_213554) diluted 1/2000 in TBS-T supplemented with 1% BSA.

The sections incubated with antibodies against ATXN3 and calbindin were incubated overnight at 4 °C and then washed three times with TBS-T (3 × 10 min). The brain sections were then incubated for 1 h at room temperature with Alexa Fluor® 594 goat anti-rabbit IgG (H + L), highly cross-adsorbed (A-11037, Life Technologies, Zug, Switzerland) diluted 1/1000 in TBS-T supplemented with 1% BSA. Finally, the brain sections were washed three times in TBS (3 × 10 min) and mounted on slides, in Vectashield supplemented with DAPI (Reactolab, Servion, Switzerland).

Finally, a goat polyclonal anti-IbaI antibody (Abcam, Amsterdam, Netherland; RRID: AB_2224402) diluted 1/500 in TBS-T supplemented with 1% BSA was added, the sections were incubated overnight at 4 °C and then washed three times with TBS-T (3 × 10 min). The brain sections were then incubated with biotinylated donkey anti-goat secondary antibody diluted 1/1000 in TBST supplemented with 1% BSA (Milan Analytica, Rheinfelden, CH; RRID: AB_2340396) for 1 h at room temperature. The brain sections were washed three times in TBS (3 × 10 min) and incubated with streptavidin-Cy3 (Milan Analytica, Rheinfelden, CH; RRID: AB_2337244) diluted 1/800 in TBST. The brain sections were mounted on slides, in Vectashield supplemented with DAPI (Reactolab, Servion, Switzerland).

### DNA and RNA extraction of cerebellar punches

Genomic DNA was extracted with the QuickExtract Kit (Lubioscience, Zurich, Switzerland) 4–6 days after the transfection of HEK293T cells, according to the manufacturer’s recommendations. For the in vivo experiment, DNA/RNA was extracted from cerebellar punch biopsy specimens with the AllPrep DNA/RNA Mini Kit (Qiagen®, Basel, Switzerland). Briefly, the collected brain punch specimens were homogenized in lysis buffer and loaded onto the DNA spin column. They were centrifuged according to the manufacturer’s instructions. The flowthrough was collected for subsequent RNA isolation, whereas the DNA was bound to the column membrane. RNA was isolated by centrifuging the flowthrough from the first step on an RNeasy spin column (Qiagen®, Basel, Switzerland). Both DNA and RNA spin columns were washed and the nucleic acids were eluted with elution buffer for DNA and RNase-free water for RNA. DNA and RNA concentrations were determined with a Nanodrop™ 2000 Spectrophotometer. DNA was stored at −20 °C and RNA at −80 °C.

### RNA levels in cerebellar punch specimens

RNA samples were converted into cDNA by reverse transcription with Superscript II (Thermo Fisher Scientific, Reinach, Switzerland) according to the manufacturer’s instructions. RNA and cDNA samples were then stored at −80 °C.

SpCas9 self-editing in MJD84.2 9 mice was analyzed at the RNA level, to avoid amplifying ssAAVs, which are not a substrate for SpCas9 [26]. The RT-qPCR reaction was performed with 7.5 ng of cDNA and primers targeting the SpCas9 (FWD: TTTTTCGCAACGGGTTTGCC; REV: AGAAGCTGTCGTCCACCTTG), by heating at 95 °C for 5 min, followed by 40 cycles of 95 °C for 10 s and 60 °C for 30 s.

The expression of the *ATXN3L* paralog was assessed by RT-qPCR on 40 ng cDNA from human neuronal progenitor cells (NPCs) and primate striatal and cerebellar punch biopsy specimens. We used the KAPA SYBR Fast qPCR master mix (Sigma-Aldrich, Buchs, Switzerland) and primers (human ATXN3L_FWD: ACTCCTGCTTCAGAACAGCCGA and REV: GCCTTTCGTGTAGGTATGAACTG; Primate_FWD: ACTCCTGCTTCGGAAGAGCCAA and REV: GCCTTTCGTGTAGATATGAACTG) according to the manufacturer’s protocol on a Rotor-Gene Q device (Qiagen, Basel, Switzerland). The cycle parameters were: 95 °C for 10 min and then 40 cycles of 95 °C for 10 s and 60 °C for 30 s.

### TIDE analysis

Human ATXN3, ATXN3L, mouse ATXN3 and SpCas9 editing efficiencies were assessed by performing tracking of indels by decomposition (TIDE) analysis on amplified gDNA or cDNA. The indel size range was set to 10–15 and the size of the decomposition window was adapted for reads of low quality or those containing repetitive sequences. The significance cutoff was set to *p* = 0.05 (https://tide.nki.nl/). Nucleic acid amplification was performed on 100 ng gDNA or 10 ng cDNA with the KAPA HiFi Hotstart kit and GC buffer (KAPA Biosystems, Labgene) according to the manufacturer’s recommendations. The primers and conditions used are described in the supplemental Materials and Methods section.

### Fluorescence-activated nuclear sorting (FANS)

The solutions for isolating cerebellar nuclei from frozen punch biopsy specimens were those described by Nadelmann and coworkers [30]. Briefly, green fluorescent protein (GFP)-positive cerebellar punch biopsy specimens were flash frozen in liquid nitrogen. For the isolation of nuclei, thawed punch specimens were transferred to 1.5 mL Eppendorf tubes containing 150 μL homogenization buffer (0.25 M sucrose, 25 mM KCl, 5 mM MgCl_2_, 0.1 mM DTT, 0.1% Triton X-100, 10 mM Tris-HCl pH 7.8, protease inhibitor cocktail (Sigma, Buchs, Switzerland), 0.2 U/µL Superase-In RNase inhibitor (Life Technologies, Zug, Switzerland), 0.4 U/µL RNasin ribonuclease inhibitor (Life Technologies, Zug, Switzerland)). The tissue was homogenized with 5 strokes in a disposable pestle and incubated on ice for 10 minutes. The homogenate was filtered through 40-µm-mesh cell strainers and the Eppendorf tubes were then washed with 100 μL homogenization buffer. Filtrates were collected and centrifuged at 500 × *g*, 4 °C for 10 min. Nuclei pellets were resuspended in 400 μL PBS, 1% BSA, 0.2 U/μL Protector RNAseIn (Sigma, Buchs, Switzerland), 4′,6-diamidino-2-phenylindole (DAPI) 1 µg/mL. Nuclei were then immediately analyzed with a FACSAria II SORP and the 70 µm nozzle. The cerebellar nuclei were sorted on the basis of DAPI staining (UV lasers) and GFP fluorescence. We resuspended 500 GFP^−^ and 500 GFP^+^ nuclei in 5 µL storage buffer from the REPLig kit for DNA analysis (Qiagen®, Basel, Switzerland) and 5000 GFP^−^ and 5000 GFP^+^ nuclei in 5 µL PBS, 1% BSA, 0.2 U/µL Protector RNAseIn (Sigma, Buchs, Switzerland) for RNA analysis.

### REPLI-g on cerebellar nuclei

Nuclear gDNA amplification (on 500 nuclei) was performed with the REPLI-g kit (Qiagen®, Basel, Switzerland) according to the manufacturer’s instructions. PCR (ATXN3) for the TIDE analysis was performed on 1/100 of the REPLI-g amplification product.

Five thousand nuclei were lysed with the RNeasy extraction kit (Qiagen®, Basel, Switzerland). RNA samples were converted into cDNA by reverse transcription with Superscript II (Thermo Fisher Scientific, Reinach, Switzerland) according to the manufacturer’s instructions. The cDNA samples were then stored at −80 °C. REPLI-g amplification was performed with 1/10 of the cDNA and Cas9-PCR for the TIDE analysis was performed with 1/100 of the REPLI-g-amplified cDNA.

### RT-qPCR on cerebellar nuclei

For quantification of the biological outcome of ATXN3 editing, we used cerebellar markers previously shown to be dysregulated in MJD84.2 mice [31, 32]: carbonic anhydrase 2 (Car2-FAM; Gene Expression Assay Thermo Fisher Scientific: Mm00501576_m1), glutamate-ammonia ligase (Glul-FAM; Gene Expression Assay Thermo Fisher Scientific: Mm00725701_1), interleukin 33 (IL33-FAM; Gene Expression Assay Thermo Fisher Scientific: Mm00505403_m1), myelin-associated oligodendrocytic basic protein (MOBP-FAM; Gene Expression Assay Thermo Fisher Scientific: Mm02745649_m1). The cycle parameters were: 95 °C for 5 min and then 40 cycles of 95 °C for 10 s and 60 °C for 30 s. Samples were normalized against the ribosomal protein L13A housekeeping gene (Rpl13a-VIC; Gene Expression Assay Thermo Fisher Scientific: Mm05910660_g1).

### Qiacuity digital PCR analysis

All analyses were performed by digital PCR in QIAcuity 24-well nanoplates with 8500 partitions (QIAcuity® digital PCR, Qiagen, Basel, Switzerland). Each reaction was set up according to the manufacturer’s instructions in a volume of 12 µL. The data were analyzed using the QIAcuity Software Suite 2.0.20 (Qiagen, Basel, Switzerland).

The deletion of ATXN3 exon 10 deletion in the cerebellum of MJD84.2 mice was quantified with 20 ng of genomic DNA. The QIAcuity EvaGreen PCR master mix (Qiagen, Basel, Switzerland), and 0.4 µM primers (CGGGACCTATCAGGACAGAG and GATCACTCCCAAGTGCTCCT) were used for the *ATXN3* gene. A QIAcuity probe PCR master mix (Qiagen, Basel, Switzerland), 0.4 µM primers and VIC-probes (TTGTGTCTCCAGTCTGCTTG, AGGTGGTGGTGGTGGTA and VIC-CCCTCTCCTGGCTCTAAATGTTGTGT-BHQ1) were used for the housekeeping gene *PCBP2*. Loaded plates were subjected to EcoRI-HF digestion for 10 min, followed by PCR as follows: initial denaturation at 95 °C for 2 min, 40 cycles of amplification at 95 °C, 60 °C, and 72 °C for 15 s each, and a final step at 40 °C for 5 min.

### Protein extraction and capillary-based immunoassay

Proteins were extracted from HEK293T in RIPA buffer (R0278, Sigma-Aldrich, Buchs, Switzerland) supplemented with a 1/200 dilution of protease inhibitor cocktail (P8340, Sigma-Aldrich, Buchs, Switzerland) and 5 µM Z-VAD-FMK (HY-16658B, Chemie Brunschwig, Basel, Switzerland), hereafter referred to as RIPA^+^ buffer. Approximately 1 × 10^6^ cells were washed in PBS and 50 μL of RIPA^+^ buffer was added to the cell pellet. The pellet was homogenized with a pellet mixer (VWR, Dietikon, Switzerland) and left on ice for 30 min. The protein extract was then centrifuged at 18,000 × *g* for 15 min at 4 °C and the supernatant containing solubilized proteins was collected into a new tube and stored at −80 °C. Protein concentration was assessed with a BCA kit (Thermo Fisher Scientific, Reinach, Switzerland) according to the recommended procedure. After dilution to a final concentration of 0.05 or 0.2 mg/mL with 0.1x SB buffer (Bio-Techne), total protein extracts were subjected to a capillary-based immunoassay on a Jess SimpleWesternTM instrument (ProteinSimple(R), Bio-Techne, Minneapolis, USA) equipped with a 12–230 kDa separation module (SM-W004) according to the manufacturer’s instructions except that we used a migration time of 28 min and an incubation period of 60 min for the primary antibody. The Replex (RP-001) and Total Protein (DM-TP01) modules were used for the sequential detection of total proteins. Ataxin3 was detected by chemiluminescence with a rabbit anti-ataxin3 antibody (Abcam ab175265, RRID: Ab_2940952, Abcam, Amsterdam, Netherland) diluted 1/50 in 0.1% Tween 20, 3% non-fat milk and ready-to-use anti rabbit-HRP (DM-001, Bio-Techne, Minneapolis, USA) in TBS.

### Image acquisition

An AxioScan.Z1 slide scanner with a ×10 objective (Zeiss, Feldbach, Switzerland) and an automated digital camera (3CCD Hitachi HV-F202SCL) was used to visualize the pattern of GFP transduction in the cerebellum of MJD84.2 mice.

All confocal images were acquired with an Olympus FV 3000 confocal microscope (Olympus, Wallisellen, Switzerland). For the co-expression studies, images were acquired with a ×20 objective (images 636.4 µm by 636.4 µm), and for assessments of the functionality of the replace vector, images were acquired with a ×60 objective. Images 212 µm by 212 µm in size were acquired with a *z*-stack of 20 µm. Acquisition parameters were kept constant for all images. For the immunofluorescence analysis in the final proof-of-principle study, images were obtained with a ×40 objective for ATXN3, calbindin, GFAP and IbaI (images 318 µm by 318 µm).

### Statistical methods

For the statistical analyses, we first tested the normality of the data distribution to determine the most appropriate statistical test. Unpaired Student’s *t* tests were used for pairwise group comparisons and nested *t* tests were used for the analysis of multiple punch biopsy specimens isolated from individual animals. One-way analysis of variance (ANOVA) was used for comparisons of more than two groups. We checked that the data were normally distributed, with equal variances, according to the requirements for ANOVA. Figures were generated with GraphPad software (GraphPad Prism version 10.0 for Mac, GraphPad Software, La Jolla California USA, www.graphpad.com). No specific method was used for sample randomization, sample-size estimation, or data inclusion/exclusion. All results are presented as the mean ±SD.

## Results

### Design and validation of sgRNAs for the induction of exon 10 deletion

**Fig. 1:**
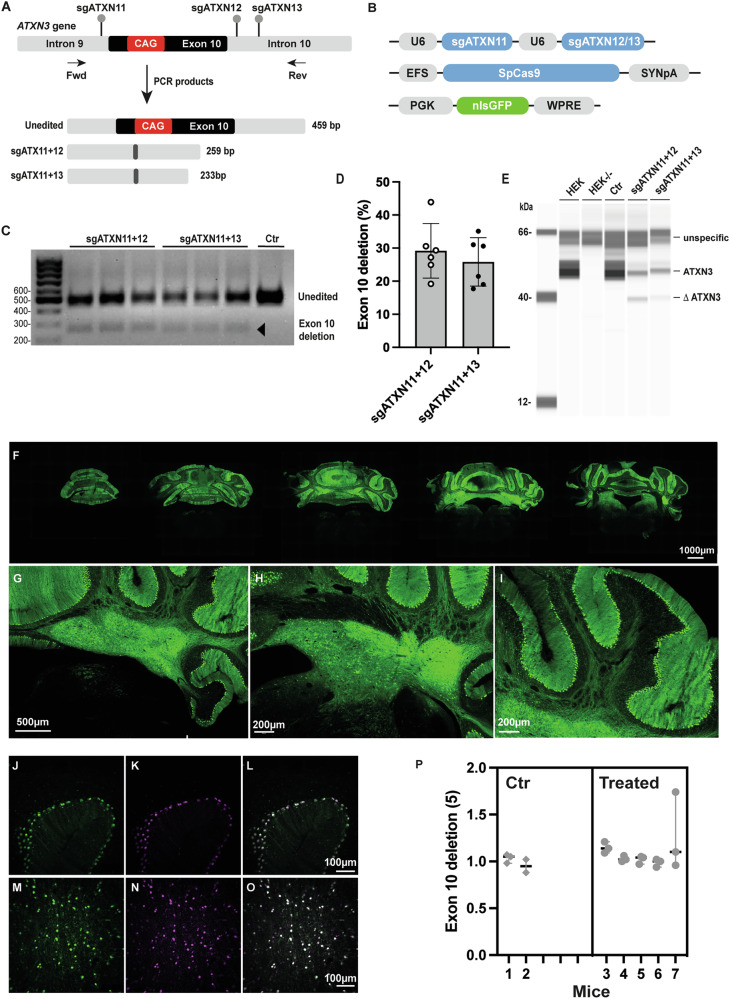
Design and screening of sgRNAs for the human *ATXN3* exon 10 deletion. **A** Three sgRNAs were designed, corresponding to intron 9 (sgATXN11) and intron 10 (sgATXN12,13). PCR was performed with forward (Fwd) and reverse (Rev) primers to amplify the targeted region of the human *ATXN3* gene. PCR products of 459–480, 259 and 233 bp were expected for the unedited gene and for exon 10 deletion with sgATXN11/12 and sgATXN11/13, respectively. **B** HEK cells were transfected with three plasmids encoding the selected sgATXN11/12 or sgATXN11/13 under the control of the U6 polymerase III promoter, the SpCas9 nuclease under the control of the short elongation factor promoter and a synthetic polyA^+^ signal, and a plasmid encoding the green fluorescent reporter gene (GFP) under the control of the mouse phosphoglycerate kinase I promoter and a woodchuck hepatitis post-transcriptional regulatory element (WPRE). HEK293T cells expressing SpCas9 and GFP were used as a negative control. **C** Representative agarose gel image demonstrating exon 10 deletion (*n* = 3/group). The deleted fragments (depicted by black arrows) are 259 bp and 233 bp in size for sgATXN11/12 and sgATXN11/13, respectively. **D** Efficiency of exon 10 deletion, as calculated from the agarose gel by dividing the intensity of the deleted fragments by the sum of intensities for all fragments. Intensities were normalized by the length of each PCR product. **E** Representative image of a capillary-based-immunoassay with the rabbit polyclonal antibody Ab175265 showing the ATXN3 protein (51–55 kDa: 3QK-10Q and 3QK-17Q) [12] in untreated and control conditions (Ctr: Cas9 only). A HEK293T clone (2-H11: HEK293T−/−) no longer expressing ataxin-3 was used to verify the specificity of the antibody. The expected loss of the full-length ATXN3 protein was observed. The production of truncated products (≈40 kDa) was visible only if the gel was overexposed. **F**–**I** Characterization of AAV2/rh.10 transduction in the cerebellum of hemizygous MJD84.2 mice. Three weeks post-injection, the diffusion of the vector was visualized by direct fluorescence. **J**–**O** Bilateral injections into the deep cerebellar nuclei (DCN) of wild-type mice were performed, AAV2/rh.10-CBA-H2B-GFP and AAV2/rh.10-CBA-H2B-mCherry. Confocal images were acquired after three weeks and showed that most cells co-expressed the two reporter genes. **P** A highly sensitive digital PCR-based (dPCR) assay was used to assess exon 10 deletion in vivo. This assay used primers amplifying of a region within exon 10, such that exon 10 deletion led to signal loss. Poly(rC)-binding protein 2 (PCBP2) amplification was used for normalization [54]. Deletion events were very rare or absent in vivo *(*CTR: *n* = 2 mice; 3 punches/animal, Treated: *n* = 5 mice; 3 punches/animal). Statistics: nested two-tailed *t* test: *P* = 0.3901, t(0.9406), df(5), median values are indicated with 95% CI.

We designed three sgRNAs with low rates of off-target binding in the human and mouse genomes (Supplementary Fig. S1D–G). These three sgRNAs recognize sequences in the introns flanking exon 10 (Fig. 1A). All sgRNAs are expressed from the U6 polymerase III promoter and a modified tracrRNA [33, 34], to maximize expression levels (Fig. 1B). The editing efficiency of individual sgATXNs was evaluated by the transient transfection of HEK293T cells (3QK10Q; 3QK17Q CAG repeats at the *ATXN3* locus [12]; data not shown). The candidates were then used to test the occurrence of *ATXN3* exon 10 deletion in a PCR-based amplification assay (Fwd and Rev primers; Fig. 1A). Amplification of the *ATXN3* locus should result in full-length amplicons of 459–480 bp, whereas the combined expression of sgATXN11/sgATXN12 and sgATXN11/sgATXN13 should induce a deletion of the corresponding genomic DNA and the production of 259 and 233 bp PCR products (Fig. 1C). Deletion efficiency reached 29.2 ± 8.3% for sgATXN11/12 and 25.8 ± 7.3% for sgATXN11/13 (Fig. 1D). Sequencing of the PCR products confirmed the presence of the expected exon 10 deletion products and another product with a 1 bp insertion (G insertion; Supplementary Fig. S1A–C). Finally, a Jess capillary-based immunoassay demonstrated the association of these deletions with the appearance of a truncated ATXN3 protein of the expected molecular weight (Fig. 1E). The specificity of the antibody was demonstrated by the absence of a signal for a HEK293T clone in which ATXN3 was inactivated by CRISPR (HEK^−/−^; Fig. 1E).

For the in vivo testing of this strategy, we used hemizygous MJD84.2 transgenic mice [28]. These animals carry the full-length human mutant *ATXN3* with 84 CAGs under the control of its native promoter (YAC-ATXN3-84Q; 250 kb) and are therefore a suitable model in which to evaluate our strategy. Multiple copies of the transgene can have a negative effect on editing efficiency [26]. We therefore quantified the absolute number of human *ATXN3* copies by digital PCR with human-specific primers. As a reference, we used genomic DNA from HEK293T cells and NPCs, and C57BL/6 mice as a negative control (Supplementary Fig. S1H). We found that MJD84.2 hemizygous (+/−) mice contained four copies of the human mutant *ATXN3* gene (Supplementary Fig. S1H). We then optimized the injection coordinates and dose of AAV2/rh.10 expressing the GFP reporter in the deep cerebellar nuclei (DCN) (Fig. 1F-I). The DNC have many connections with the cerebellar cortex and are the major terminal projection sites of Purkinje cells. Moreover, DCN also connect with several other central nervous system sites that are also affected in SCA3, such as the spinal cord, pons and medulla [35]. Thanks to this unique cerebellar anatomy and the retrograde transport properties of AAV, local delivery to the DCN resulted in a widespread distribution throughout the cerebellum (Fig. 1F–I). Given the relatively large size of the SpCas9 and the limited capacity of AAV vectors, the gene editing machinery was delivered by separate vectors requiring co-expression for efficient gene editing. The co-delivery of AAV2/rh.10 expressing GFP or mCherry resulted in colocalization in most of the transduced cells in the cerebellum, including the cerebellar lobes and the Purkinje cell layer (Fig. 1J–0).

We then evaluated the efficiency of *ATXN3* exon10 deletion in MJD84.2 transgenic mice. We injected AAV2/rh.10 vectors expressing the SpCas9, sgATXN11/12 or sgATXN11/13, and a GFP reporter gene expressed in the nucleus (nlsGFP) into the mouse cerebellum to visualize the transduced cells (Fig. 1B). The animals were killed four weeks after the injection. Cerebellar punch specimens were collected from the nlsGFP-positive areas for genomic DNA extraction. Quantification of the deletion event in a digital PCR-based assay (Supplementary Fig. S1I–J) showed a very low level of exon 10 deletion events (Fig. 1P), consistent with recent studies highlighting the challenges of dual-sgRNA strategies in the brain [25, 36].

### Design and validation of sgRNAs for the induction of exon 9 truncation

**Fig. 2:**
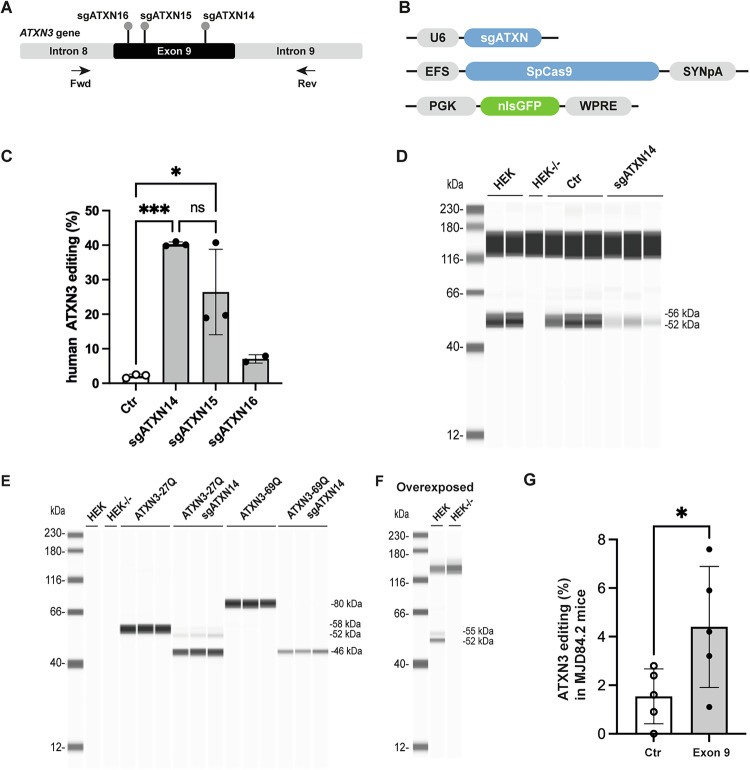
Exon 9 truncation. **A** Scheme showing the location of sgATXN14/15/16 binding sites in exon 9 of the *ATXN3* gene. The black arrow indicates the primers used for TIDE analysis. **B** Plasmids used in HEK 293 T cell transfection experiments to assess sgATXN functionality. After three days, the DNA was extracted, PCR was performed on the region of interest and the sequencing files generated were analyzed by TIDE. **C** Graph showing the editing efficiency of the various sgRNAs. Control, sgATXN14 and sgATXN15 (*N* = 1 independent experiment, *n* = 3 samples); sgATXN16 (*N* = 1 independent experiment, *n* = 2 samples). Results are plotted as the mean ± standard deviation and analyzed by one-way ANOVA followed by Brown-Forsythe post-hoc test: F(3,7) = 20,05, *** *p* = 0.0008. The displayed data are normalized against transfection efficiency (GFP). **D** Representative image of a capillary-based immunoassay showing the loss of full-length ATXN3 (52–56 kDa) from treated samples (sgATXN14). We were unable to visualize the presence of a truncated protein, probably because HEK293T cells express *ATXN3* only weakly. **E** We overcame this limitation by transfecting HEK293T cells with plasmids encoding human ATXN3-27Q (*n* = 3) or ATXN3-69Q (*n* = 3). In both cases, a truncated product (46 kDa) was clearly visible in the treated samples. **F** Overexposure of the gel made it possible to visualize the endogenous human ATXN3. **G** TIDE analysis performed 3 weeks post-injection, on the cerebellum of MJD84.2 mice (*n* = 5/group), revealed very low rates of exon 9 editing in vivo. Statistics: *t-*test, *P* = 0.0477, t(2.336), df(8).

As an alternative, we decided to generate truncated ataxin-3, with a single sgATXN targeting exon 9 of the human ATXN3 *gene* (Fig. 2A, B, Supplementary Fig. S1D–G). Exon 9 is relatively small and the CRISPOR webtool identified only three guide sequences. The editing efficiency of these guide sequences, sgATXN14, sgATXN15 and sgATXN16, was measured in HEK293T cells by TIDE analysis three days after transfection. Editing rates of 40.3 ± 0.6% and 26.5 ± 12.3% were obtained with sgATXN14 and sgATXN15, respectively, whereas sgATXN16 was inefficient, with an indel rate of only 7.1 ± 1.2% (Fig. 2C). The Jess capillary-based immunoassay for endogenous ATXN3 detected no truncated product (Fig. 2D), probably due to the low levels of *ATXN3* expression in HEK293T cells and/or the short half-life of the truncated protein. We overcame this limitation through the use of plasmids encoding a human ATXN3-27Q or ATXN3-69Q (Fig. 2E), which yielded a truncated protein of 46 kDa. As expected, the endogenous ATXN3 (52 kDa) was visible only if the image was overexposed (Fig. 2F).

Based on these data, we selected sgATXN14 for in vivo evaluation (Fig. 2A). The indel profile indicated the probable insertion of a premature stop codon at the 3’-end of exon 9, resulting in a 286-amino acid protein fragment. Validation was performed in MJD84.2 mice as for the exon 10 deletion, but TIDE analysis showed that editing levels were close to background, at only 1.4–5.3 ± 1.2% (Fig. 2G).

### Design and validation of sgRNAs for the ablate strategy

**Fig. 3:**
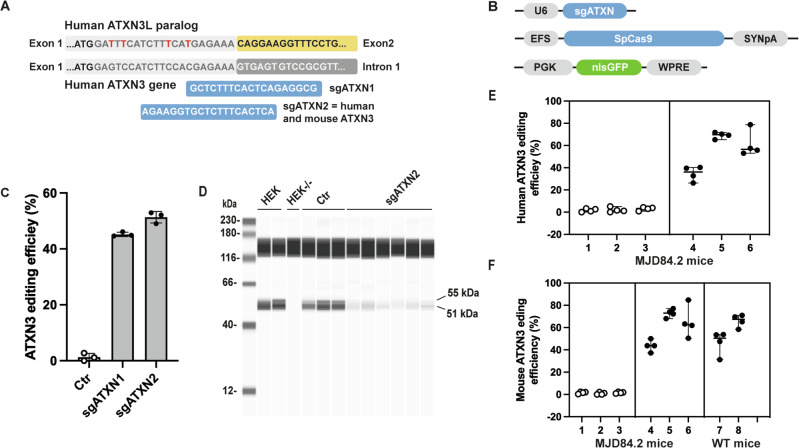
Ablate. **A** Scheme showing the location of sgATXN1 and sgATXN2 binding to exon 1 of the human *ATXN3* gene and the human *ATXN3L* paralog. **B** Plasmids used to transfect HEK 293T cells to assess sgATXN functionality. **C** In vitro editing efficiencies, 3 days after the transfection of HEK293T cells (*n* = 3/group). **D** Capillary-based immunoassay showing the expected loss of the full-length ATXN3 protein in samples treated with sgATXN2 (Ctr: *n* = 4, Treated with sgATXN2: *n* = 6). **E**, **F** In vivo editing efficiencies (*n* = 3 mice/group, 4 punches/animal). **E** Human mutant ATXN3-84Q editing levels reached 71.7% in GFP^+^ punch specimens from the cerebellum. Statistics: nested two-tailed *t*-test: *P* = 0.0071, t(5.067), df(4), median values are shown with 95% CI. **F** Similar editing efficiencies were obtained for the endogenous mouse WT-ATXN3 gene in MJD84.2 and WT animals. Statistics: nested one-way ANOVA: *P* = 0.0027, F(2,5) = 24.08, median values are shown with 95% CI.

**Fig. 4:**
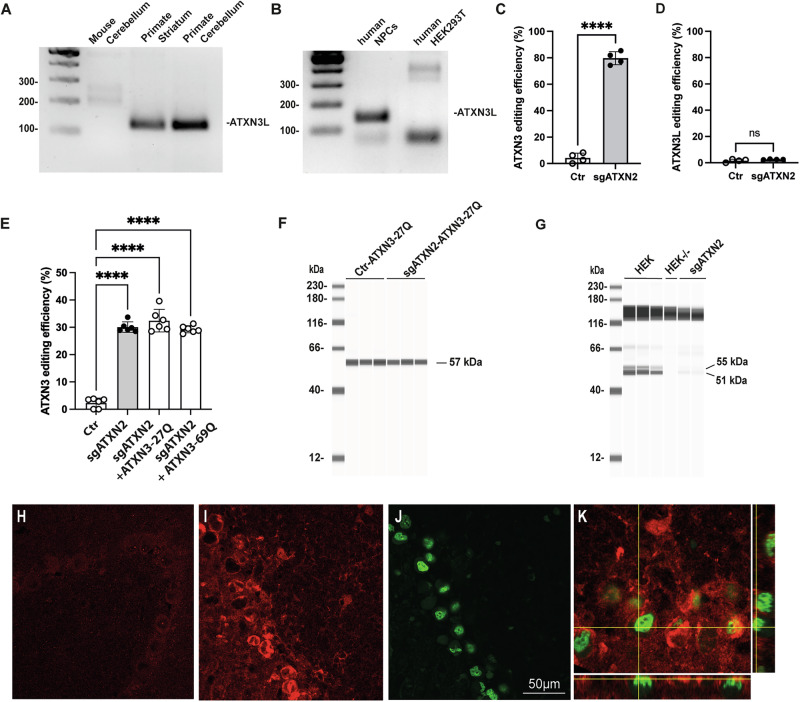
Validation of the replace vector. **A** RT-PCR showing the expression of *ATXN3L* mRNA in the striatum and cerebellum of primates (*n* = 2) and its absence in the cerebellum of mice (*n* = 1). **B** *ATXN3L* expression in human neuronal progenitor cells (NPCs) and absence in HEK293T cells. **C** Editing experiment in NPCs showing the inactivation of ATXN3 at 79.7 ± 3.9% and **D** CRISPR-resistance of the ATXN3L paralog (Ctr: *n* = 4, Treated with sgATXN2: *n* = 4). Statistics: Results are plotted as the mean ± standard deviation and were analyzed by *t*-test: ATXTN3L: ns, t(1,645), df(6)). ATXN3: ****p < 0.0001, t(24), df(6). **E** Endogenous ATXN3 editing efficiency was not affected by the expression of the replace vector (ATXN3-27Q) or the expression of a mutant ATXN3 (ATXN3-69Q) in HEK293T cells. Statistics: Results are plotted as the mean ± standard deviation and were analyzed by one-way ANOVA followed by Brown-Forsythe post-hoc test. F(3,20) = 190,6, ****p < 0.0001. **F** Capillary-based immunoassay showing that the replace vector is resistant to treatment with sgATXN2 (Ctr: *n* = 3, Treated with sgATXN2: *n* = 3), whereas (**G**) the e*n*dogenous ATXN3 is efficiently inactivated in the same samples. **H**–**K** Representative confocal images from coronal brain sections showing robust expression of the replace vector in the cerebellum of wild-type mice. **H** Control, uninjected animal. **J** Transduced cells are identified with the fluorescent reporter gene GFP, in green, **I** Expression of the human replace ATXN3-27Q vector is shown in red. **K** Confocal images and orthogonal projections showing the presence of ATXN3-27Q in GFP-positive cells.

In this final strategy, we designed sgRNAs for the inactivation (ablate) of *ATXN3* but not its human *ATXN3L* paralog (multiple mismatches; Fig. 3A, B, Supplementary Fig. S1D–G). We selected sgATXN1 and sgATXN2, targeting a region close to the translation start site of the human *ATXN3* gene (Fig. 3A). A TIDE analysis of transfected HEK293T cells revealed high editing efficiencies for both sgRNAs (Fig. 3C). We selected sgATXN2 for subsequent analyses, because it led to the generation of a premature stop codon at the 5’-end of exon 2, displayed full sequence identity with the mouse *ATXN3* gene and had more mismatches with the *ATXN3L* paralog (CRISPR-resistant) (Fig. 3A). A Jess capillary-based immunoassay confirmed the loss of the ATXN3 protein from treated HEK293T cells (Fig. 3D). A recent study revealed an association of ataxin-3 with the transcription-coupled nonhomologous end-joining (TC-NHEJ) process through interaction with polynucleotide kinase 3’-phosphatase (PNKP) [37]. In addition to affecting SCA3 pathogenesis, this might also compromise therapeutic efficacy. We therefore assessed the impact of mutant ATXN3-69Q in HEK cells [38]. Endogenous human ATXN3 editing was not affected by the presence of the mutant ATXN3-69Q (Fig. 4C). Finally, the treatment of MJD84.2 mice (Fig. 3B) in vivo led to the robust editing efficiency of both human (55 ± 18%; Fig. 3E) and mouse (58 ± 14%; Fig. 3F) *ATXN3* genes, with the highest indel rates reaching up to 71.7% and 77%, respectively. Editing efficiency was not affected by the number of copies of the *ATXN3* gene in MJD84.2 (2 copies of WT mouse ATXN3 and 4 copies of human mutant ATXN3) or C57BL/6 (2 copies of WT mouse ATXN3) mice (Fig. 3F).

### Use of the ATXN3-27Q vector to mimic *ATXN3L* replacement in mice

Limited data are available concerning the expression of the *ATXN3L* paralog in brain cells. We therefore first confirmed the expression of this paralog in NPCs and cerebellar and striatal samples from a non-human primate (NHP), by RT-qPCR analysis with specific primers (Fig. 4A, B). Semi-quantitative comparison indicates that the level of expression of the paralog is lower than the ATXN3 both in primates and NPC (7,9 fold and 22,7 fold, respectively). CRISPR-resistance of the endogenous human ATXN3L paralog was demonstrated in NPCs (Fig. 4C, D). No editing of the ATXN3L paralog was observed while the endogenous ATXN3 gene editing reached 79.7 ± 3.9%. The *ATXN3L* paralog is not present in mice, we therefore developed a CRISPR-resistant replacement vector encoding a wild-type human ATXN3-27Q (Supplementary Fig. S2A). We checked for CRISPR resistance in HEK293T cells. The endogenous human *ATXN3* gene was edited equally well in the presence and absence of the replacement vector (Fig. 4E, Supplementary Fig. S2B). PCR amplification with the replace vector and endogenous ATXN3L paralog with specific primers and sequencing of the region of interest demonstrated the absence of indels (Supplementary Fig. S2C–F). A Jess capillary-based immunoassay further demonstrated the CRISPR-resistance of ATXN3-27Q protein (Fig. 4F). As expected, there was a significant decrease in ATXN3 protein expression in these samples (Fig. 4G, data not shown). For further validation of the replacement vector, we co-injected ATXN3-27Q and the nuclear GFP reporter gene into the DCN of wild-type mice (Fig. 4H–K). Three weeks after injection, coronal cerebellar slices were obtained and stained with anti-ataxin-3 antibody for visualization by confocal microscopy. High levels of ATXN3-27Q were observed in GFP-positive transduced cells (Fig. 4H–K).

### KamiCas9 ablate-and-replace proof-of-principle study in MJD84.2 mice

**Fig. 5:**
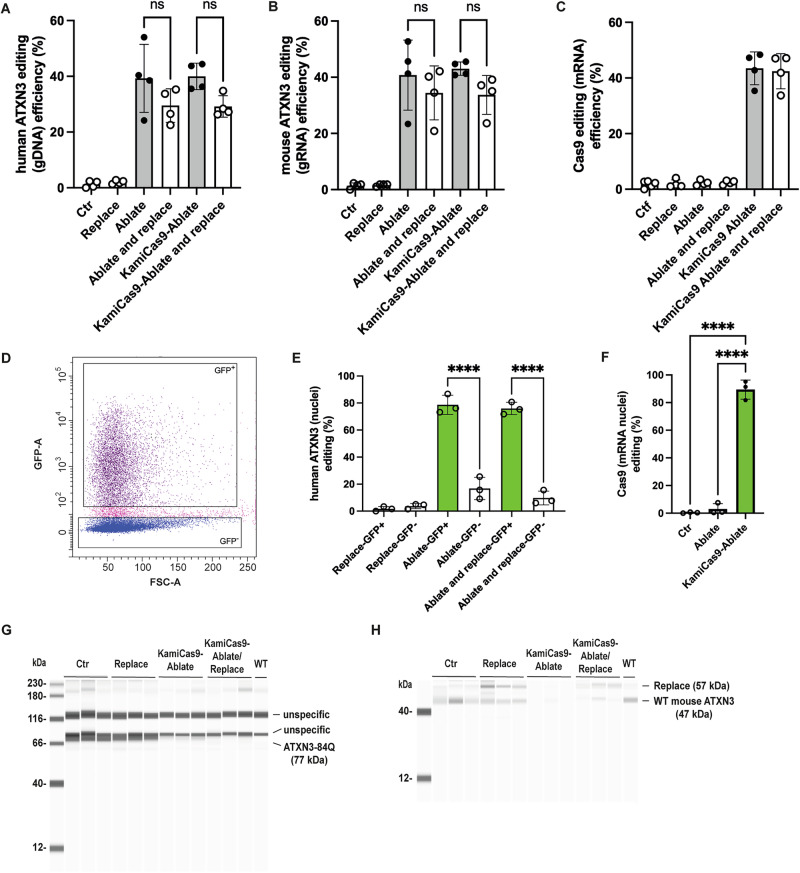
KamiCas9 ablate-and-replace strategy in MJD84.2 mice. **A** Human ATXN3-84Q editing in the cerebellum of Ctr (*n* = 4 mice) and treated animals (*n* = 4 mice/group). In all treated animals, robust editing of the human *ATXN3* was observed, with no difference between ablate and ablate/replace strategies, or between KamiCas9 ablate and KamiCas9 ablate/replace strategies. **B** Similar results were obtained for editing of the endogenous mouse WT ATXN3. **C** Cas9 editing (mRNA level) confirmed the self-inactivation of the system. **D** FANS analysis of nuclei prepared from mouse cerebellum. Representative image of the sorting of GFP^+^/GFP^−^ cerebellar nuclei. **E** TIDE analysis of GFP^+^/GFP^-^ sorted nuclei revealed that ATXN3 editing levels reached 75.6 ± 4.5% (ablate) and 78.6 ± 7.1% (ablate/replace) in GFP^+^ nuclei, twice the editing efficiencies measured in cerebellar punch specimens. **A** Statistics: Results are plotted as the mean ± standard deviation and were analyzed by one-way ANOVA followed by Tukey’s multiple comparison test. ****: *p* < 0.0001. **F** We also observed an enrichment of Cas9 editing, demonstrating excellent self-inactivation of the system at two months. Statistics: Results are plotted as the mean ± standard deviation and were analyzed by one-way ANOVA followed by Brown-Forsythe post-hoc test. F(2, 6) = 363,7, ****p < 0.0001. **G** Capillary-based immunoassay of GFP-positive cerebellar punch specimens showing the expected loss of expression of ATXN3-84Q (77 kDa) in the KamiCas9 ablate and KamiCas9 ablate/replace groups (*n* = 3 animals/group, *n* = 1 punch specimen/animal). **H** For visualization of the replace vector (ATXN3-27Q: 57 kDa) and the WT mouse ATXN3 (47 kDa), the gel was overexposed. The inactivation of the WT mouse ATXN3 (47 kDa) is clearly visible in the ablate samples and, to a lesser extent, in the ablate-and-replace samples. Finally, the replacement vector was expressed, as expected, in the corresponding groups.

**Fig. 6:**
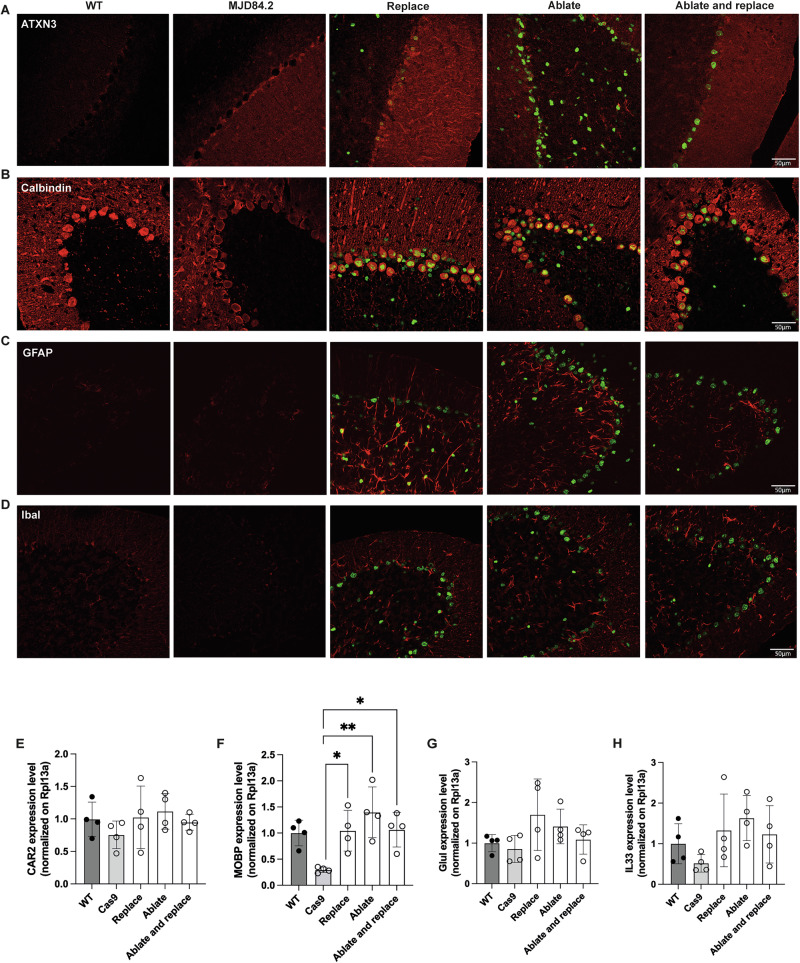
Gene editing is well tolerated in MJD84.2 mice. **A**–**D** Immunofluorescence staining for ATXN3 (red) (**A**) showing the overexpression of human ATXN3 in MJD84.2 mice and in the replace groups (transduced cells identified with the H2B-staygold fluorescent reporter) relative to WT animals. In treated mice, a significant decrease in ATXN3 levels was observed in the ablate animals and, as expected, a restoration of human wild-type ATXN3 was visible in the ablate-and-replace group. Calbindin expression (red) (**B**) was unaltered in all cases, and GFP expression showed that the Purkinje cells were transduced. A slight increase in GFAP (red) (**C**) and IbaI (red) (**D**) expression was observed in the treated animals. **E**–**H** Therapeutic efficacy assessed on cerebellar punch specimens (*n* = 4 mice/group). The levels of CAR2 (**E**), MOBP (**F**), GLUL (**G**) and IL33 (**H**) expression measured by RT-qPCR were normalized against the housekeeping gene Rpl13a. The results are plotted as the mean ± standard deviation and were analyzed by one-way ANOVA followed by Tukey’s multiple comparison test. **p < 0.01, *p < 0.05.

For the final in vivo proof-of-principle studies of the ablate-and-replace strategy, we used an optimized self-inactivating kamiCas9 (Supplementary Fig. S3A–D) and an H2B-staygold fluorescent reporter gene to identify transduced cells. H2B-staygold is a photostable GFP derived from the jellyfish *Cytaeis uchidae* [39] linked to the human histone H2B sequence to promote its nuclear localization [40]. This reporter can be used to facilitate fluorescence-activated nuclear sorting (FANS). The mice were killed two months after surgery, and cerebella punch specimens were collected from the transduced area. We evaluated cas9 only (Ctr), replace, constitutive ablation or KamiCas9 and constitutive ablate-and-replace or KamiCas9 strategies (Fig. 5A). Editing efficiency was similar between: a) human mutant-ATXN3 and mouse WT-ATXN3, b) constitutive and kamiCas9 conditions and c) ablate and ablate-and-replace strategies (Fig. 5A, B). High levels of Cas9 editing, and therefore self-inactivation were observed in the KamiCas9 groups (Fig. 5C). Additional punch specimens were used for the Jess capillary assay, which demonstrated the loss of the human mutant (77 kDa) (Fig. 5G) or mouse *ATXN3* (47 kDa) (Fig. 5H) and the expression of the replace vector (57 kDa) (Fig. 5H). Immunofluorescence staining of cerebellar sections confirmed these data (Fig. 6A).

Finally, we performed FANS to characterize the editing in transduced cells in greater detail. A TIDE analysis of GFP^+^ nuclei revealed editing rates of 78 ± 7.1% for ATXN3 and 89.4 ± 6.9% for Cas9 (Fig. 5D–F). These data demonstrate the high performance of our editing system despite the presence of four copies of the human *ATXN3* gene and the massive self-inactivation of the SpCas9 nuclease in vivo.

### Analysis of cerebellar markers

Calbindin D-28K immunostaining was used to examine Purkinje neurons in C57BL/6 and MJD84.2 mice. Normal morphology was observed in all conditions (Fig. 6B), with a mild and variable loss of calbindin staining sometimes observed in MJD84.2 mice (Fig. 6B). Calbindin expression in all treated groups was similar to that in WT animals. Immunofluorescence analysis revealed a modest increase in the levels of the astrocyte marker GFAP (Fig. 6C) and the microglial marker IbaI (Fig. 6D) in animals treated with AAV2.rh10-KamiCas9.

Several studies have reported transcriptional changes in the cerebellum of MJD84.2 mice [31, 32]. We therefore investigated the response of cerebellar markers to gene editing treatment (Fig. 6E–H). We selected Car2, MOBP, GluI and IL33 for analysis. As we used relatively young hemizygous mice, the downregulation of these cerebellar markers in the Cas9-only MJD84.2 control mice was modest, reaching statistical significance only for MOBP (Fig. 6F). In all treated groups, the level of expression was equivalent to that in wild-type animals. Overall, these data indicate that KamiCas9 editing is well tolerated and can induce SCA3-specific transcriptional rescue.

## Discussion

We developed editing strategies suitable for use in all SCA3 patients by exploiting two unique properties of the ATXN3 protein: the natural occurrence of truncated but functional ATXN3 proteins lacking the third IUM domain and polyglutamine repeats, and the presence of a CRISPR-resistant *ATXN3L* paralog in humans.

We screened various sgRNA candidates in HEK293T cells and validated them in MJD84.2 mice, the only transgenic model available in which the full-length human *ATXN3* gene is expressed [28]. The cerebellum plays a key role in SCA3 pathogenesis. Changes in Purkinje cell pattern and atrophy have been reported in both mouse models [41, 42] and SCA3 patients [29]. We maximized vector transduction in a large structure (45–52 mm^3^) containing a high proportion of neurons (87% neurons according to the Blue Brain Atlas (https://bbp.epfl.ch/nexus/cell-atlas/)), by making use of cerebellar connectivity and performing intracerebral injections into the deep cerebellar nuclei (DCN). We selected AAV2/rh.10, which had high levels of high diffusion and retrograde transport properties, for this purpose [35, 43].

Despite the high levels of exon 10 deletion observed in HEK293T cells, deletion rates were low in the cerebellum of MJD84.2 mice. Interestingly, ineffective deletions have also been reported for dual sgRNA strategies in spinocerebellar ataxia type 2, Duchenne muscular dystrophy and Huntington’s disease [25, 36, 44]. This lack of deletion efficiency may be due to asynchronous cleavage by the two sgRNAs in vivo [36]. If this is indeed the case, then a strategy targeting exon 9 with a single sgRNA would be expected to solve this problem. However, this was not found to be the case, suggesting that chromatin structure may instead interfere with gene editing in vivo [45–47]. Two studies have shown that CAG expansions alter chromatin structure in polyglutamine disorders [48, 49]. Further evidence for an effect of chromatin structure is provided by the high rates of gene editing achieved with two sgRNAs targeting *ATXN3* exon 1, a region of the gene far away from the CAG repeats.

The ablate-and-replace strategy is based on the presence of an *ATXN3L* paralog in primates and humans. Analyses of the human genome have predicted that at least 15% of human genes are duplicated [50]. Interestingly, paralogs have been reported for SCA1, 2, 3, 6, 7,17 and DRPLA [18]. The *ATXN3* gene has two paralogs: *ATXN3L* on chromosome 22, resulting from an RNA-based duplication that leads to the insertion of reverse-transcribed mRNAs into the genome, and *ATXN3L2* on chromosome 8, which has a premature stop codon likely to produce an inactive pseudogene [18]. *ATXN3L* is particularly attractive as a potential endogenous replacement gene because it has no introns and CAG expansions are prevented by the presence of a GAA break in the repeats. Sequencing data suggest that *ATXN3L* is expressed in the brain and that its Josephin domain has deubiquitinating activity [51–53]. We further characterized *ATXN3L* by performing RT-qPCR analysis, which demonstrated that *ATXN3L* is expressed in the striatum and cerebellum of *Macaca fascicularis* and in human NPCs although at a lower level than the ATXN3. This level could be sufficient given that the Josephin domain of ATXN3L exhibits higher enzymatic activity than ATXN3 [53], and the deubiquitinating activity (DUB) is likely redundant given the lack of phenotype in ATXN3 knockout mice.

To corroborate this hypothesis, we mimicked the genetic landscape of SCA3 patients as closely as possible by developing a CRISPR-resistant replacement vector for mouse experiments. We showed that the wild-type *ATXN3* cDNA in this vector was expressed in the cerebellum of WT and MJD84.2 mice. For the ablate strategy, we selected an sgRNA (sgATXN2) targeting a region located at the junction of exon 1 and intron 1. This sequence is conserved in humans and mice but partially absent in the replace vector and the human *ATXN3L* paralog. In hemizygous MJD84.2 animals, the use of this sgRNA led to editing of the four copies of the mutant human *ATNX3* gene and the two copies of the wild-type mouse *ATXN3* gene, with conserved expression of the CRISPR-resistant replacement vector. TIDE analysis of cerebellar punch specimens showed that 55.0 ± 16.9% and 58.8 ± 14.2% of the mutant human and mouse WT *ATXN3* genes, respectively, were edited, despite the presence of multiple copies of the target sequence. Data from MJD84.2 homozygous mice suggests that reducing the transcription level of mutant ATXN3 by 50% prevents the accumulation of aggregates in vulnerable brain regions and restores motor function [6]. This further supports the potential of our ablate-and-replace strategy.

In the final proof-of-principle study, we assessed the constitutive and kamiCas9-ablate or ablate-and-replace strategies. Once again, high levels of editing were observed for the human and mouse *ATXN3* genes in all cases and Jess capillary assays confirmed the loss of the human mutant ATXN3 protein. We assessed treatment efficiency further by evaluating editing in GFP^+^ nuclei corresponding to transduced neurons. Human *ATXN3* editing levels in GFP^+^ cells reached 78±7.1%, with a *Cas9* self-inactivation level of 89.4 ± 6.9%. Together, these data demonstrate the potency of the KamiCas9 system for inactivating the *ATXN3* gene in vivo.

The main objective of this study was to establish the level of KamiCas9 editing. We therefore used MJD84.2 hemizygous mice to minimize the copy number of the *ATXN3* gene. Consequently, neuropathological and functional outcomes were limited, with no loss of neurons in the DCN and no motor phenotype in these animals. For further validation of this approach, a long-term study in SCA3 transgenic mice and additional data for primate and human SCA3-NPSc would be required. However, RT-qPCR and immunofluorescence analyses of cerebellar markers strongly suggest that the ATXN3 ablate-and-replace strategy is well tolerated, with no overt signs of cerebellar toxicity and potential rescue of the transcriptional signature.

## Supplementary information

Supplemental material

## Data Availability

The datasets generated and/or analyzed during this study are available from the corresponding author on reasonable request.
