# Supplementary material for: Gene editing for Spinocerebellar ataxia type 3 taking advantage of the human ATXN3L paralog as replacement gene
Source: Gene Ther. 2025 Jul 28;32(5):462–74. doi: 10.1038/s41434-025-00557-2 (PMC12518135; doi:10.1038/s41434-025-00557-2)
Supplement: Supplementary file 1 — Supplemental material [file 41434_2025_557_MOESM1_ESM.docx]

**Supplemental data**

**Gene editing strategy for spinocerebellar ataxia type 3 taking advantage of ATXN3L paralog as replacement gene**

Margareta Rybarikova^1,2^, Maria Rey^1,2^, Ed Hasanovic^1,2^, Mélanie Sipion^1,2^, Lukas Rambousek^3^, and Nicole Déglon^1,2^

^1^Lausanne University Hospital (CHUV) and University of Lausanne (UNIL), Department of Clinical Neurosciences (DNC), Laboratory of Cellular and Molecular Neurotherapies, Lausanne, Switzerland. ^2^Lausanne University Hospital (CHUV) and University of Lausanne (UNIL), Neuroscience Research Center (CRN), Laboratory of Cellular and Molecular Neurotherapies (LCMN), Lausanne, Switzerland.

^3^Lausanne University Hospital (CHUV) and University of Lausanne (UNIL), Department of Clinical Neurosciences (DNC), Neurorestore

**Supplementary Figures and Tables**

**Supplementary Figure 1: (A)** Localization of sgATXN3 11, 12 and 13 sites in the human ATXN3 gene. **(B-C)** Sequencing of the delete product for sgATXN11/12 (**B**) and sgATXN11/13 (**C**). The blue lines indicate the expected cleavage sites. The major product corresponds to the expected exon 10 deletion sequences and minor products contain an extra nucleotide (G) in both sgATXN11/12 and sgATXN11/13 sequences, probably due to a double-strand break one nucleotide downstream from the expected cleavage site of sgATXN11. (**D-G**) The potential off-target (OT) sites for each sgATXN candidate were identified by *in silico* analysis with the CRISPOR online tool. OT sites were considered if they were flanked by NGG, NAG or NGA motifs. Results of analyses searching for OT sites with up to 3 mismatches in the human (**D**) and mouse (**E**) genomes. Localization of the OT sites in exons, introns or intergenic regions of the human (**F**) or mouse (**G**) genomes. (**H**) A digital PCR assay was used to measure ATXN3 gene copy number in MJD84.2 mice; genomic DNA from HEK293T cells and HD-derived neuronal precursor cells (HD-NPCs) was used as an internal standard containing two copies of the *ATXN3* gene. In addition, genomic DNA from wild-type (WT) mice was used as a negative control, because only human ATXN3 was targeted by the probe and primers used (linked to Figure 1). (**I-J**) Absolute quantification of *ATXN3* gene copy number in MJD84.2 mice by digital PCR. A Taqman assay (FAM/VIC probes) with primers targeting the human *ATXN3* gene and the *PCBP2* reference gene were used. We first performed spiking experiments with 80 and 100 ng WT/MJD84.2 gDNA (ratio 0:100, 75:25, 50:50, 25:75, 100:0 WT/MJD84.2) to validate the assay.

**
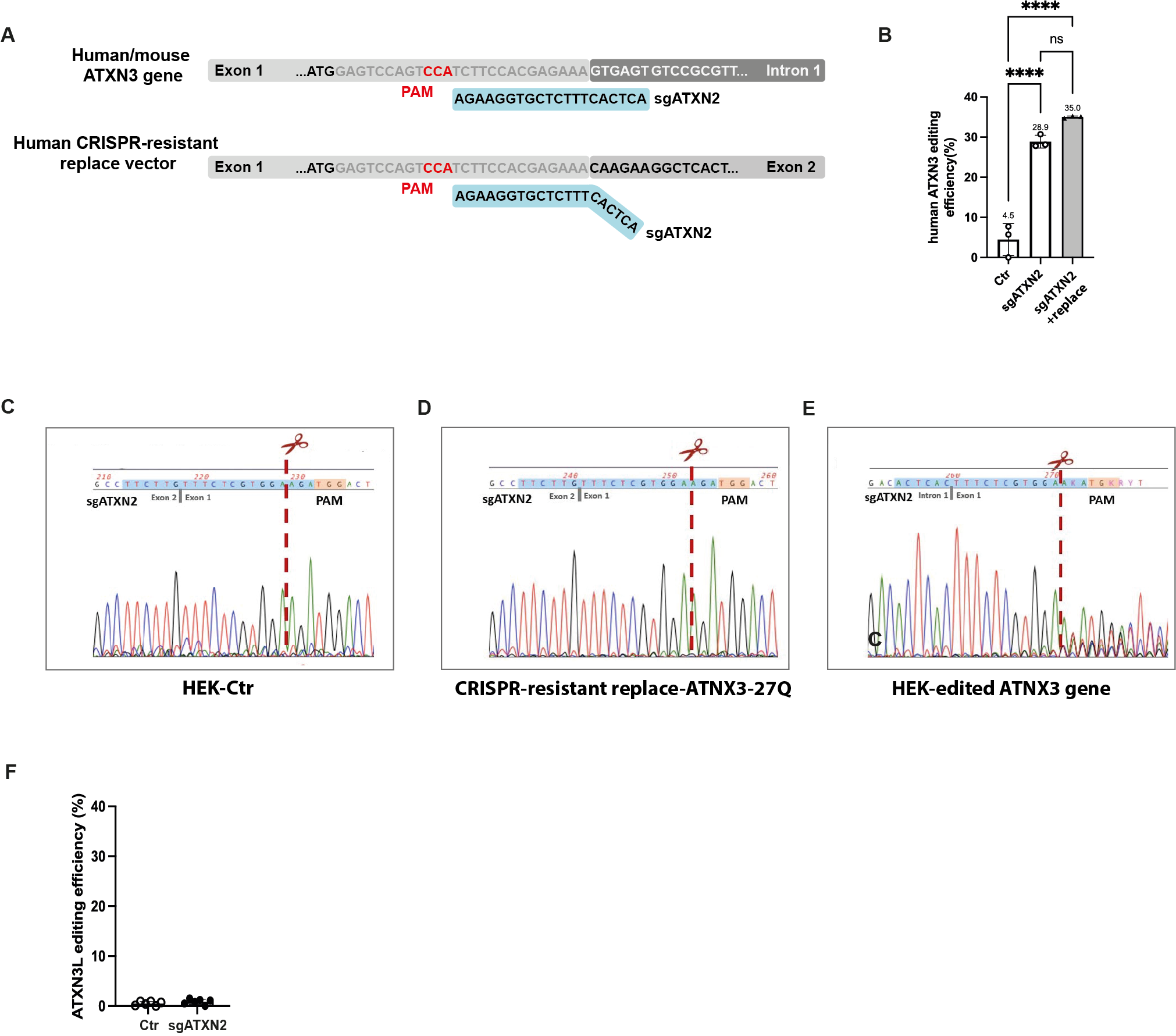
**

**Supplemental Figure 2: Validation of the replace vector. (A)** Scheme showing the target location of sgATXN2 in the human and mouse *ATXN3* genes and the CRISPR-resistant replace vector (linked to Figure 4). The translation start site in exon 1 is depicted in black. The corresponding PAM site is indicated in red. (**B**) Editing efficiency for the endogenous human *ATXN3* in the presence of sgATXN2 or sgATXN2 and the replace vector (*n* = 3). Results are plotted as the mean ± standard deviation and were analyzed by one-way ANOVA followed by **Tukey’s** post-hoc test. ****: *p* < 0.0001,***: *p* < 0.001, **: *p* < 0.01, *: *p* < 0.05. (**C**) Chromatogram comparison after sequencing, to determine whether the replace vector was CRISPR-resistant. The sgATXN2 sequence and PAM were aligned with the matching part of exon 1 of *ATXN3* from HEK293T control cells. The sequence was completed with nucleotides from exon 2 of *ATXN3* to obtain a 20 nt sequence including a false sgATXN2 sequence. (**E**) A sample with endogenous *ATXN3* edited by sgATXN2 was used for comparison (*n* = 3). (**D**) The sequence from the replace vector was analyzed to confirm the absence of significant editing (*n*=3). (**F**) ATXN3L-specific primers demonstrate the absence of editing of the endogenous human ATXN3L paralog in HEK293T cells (*t*-test: *P*=0,2287; t(1,282), df(10)).

**Supplemental Figure 3: Optimization of KamiCas9. (A)** Scheme of the plasmid constructs used for HEK293T cell transfection (linked to Figure 5). The various modifications (U6 promoter, optimized Tracr and presence of the H2B-staygold reporter cassette) are indicated in green. (**B**) Graph showing *ATXN3* editing efficiency. HEK293T cells were transfected and DNA extracted 4 days post-transfection. The region of interest was amplified by PCR and sequenced. The indel rate was quantified by TIDE. (**C**) Cas9 editing efficiency (*n*= 3). Data are plotted as the mean ± standard deviation and were analyzed by one-way ANOVA followed by Tukey’s multiple comparison test. ****: *p* < 0.0001; ***: *p* < 0.001; **: *p* < 0.01; *: *p* < 0.05. (**D**) Analysis of the indel profile demonstrating that most of the edited sequences contain a 1 bp (C or G) insertion leading to inactivation of the Cas9 gene (premature stop codon). (**E**) Finally, a FACS analysis of GFP-positive nuclei (FANS) demonstrated that editing efficiency reached 89.4±6.9% in transduced cells. Data are plotted as the mean ± standard deviation and were analyzed by one-way ANOVA followed by Tukey’s multiple comparison test. ****: *p* < 0.0001; ***: *p* < 0.001.

**Materials and Methods**

**Plasmid production**

Plasmids expressing fluorescent reporter genes: The SIN-PGK-GFP-WPRE plasmid encoding the green fluorescent protein (GFP) in a SIN lentiviral backbone (SIN-cPPT-PGK-WPRE) has been described elsewhere ^[1](#_ENREF_1)^. The H2B-GFP plasmid was a gift from Geoff Wahl (Addgene; http://n2t.net/addgene:11680; RRID: Addgene_11680). This plasmid contains a GFP reporter gene fused to human histone 2B (H2B, 126aa) to label the nucleosome core without disturbing the cell cycle ^[2](#_ENREF_2)^. The H2B-GFP fragment was transferred into the pENTR4 vector (Life Technologies, Zug, Switzerland) to produce the entry plasmid, pENTR4-H2B-GFP. An LR reaction was performed with the destination plasmid, pAAV2ss-CBA-Gateway-WPRE-bGH ^[3](#_ENREF_3)^ to generate pAAV2ss-CBA-H2B-GFP-WPRE-bGH. The plasmids encoding GFP (pAAV2ss-CBA-GFP-WPRE-bGH), or a nuclear GFP (pAAV2ss-CBA-AcGFPnuc-WHV-bGH) have been described elsewhere ^[3](#_ENREF_3)^. We ordered a 1050 bp DNA fragment (Geneart, Thermo Fisher Scientific, Zug, Switzerland) encoding the highly photostable and bright green fluorescent protein staygold ^[4](#_ENREF_4)^ fused to H2B. This fragment was used to generate pAAV2ss-CBA-H2B-staygold-SYNpA. It was also inserted into the pENTR4-CMV vector to generate pENTR4-CMV-H2B-staygold. An LR reaction was performed with the destination plasmid pAAV2ss-Gateway-SYNpolyA containing the gateway cassette and a synthetic polyadenylation site ^[5](#_ENREF_5)^ to generate pAAV2ss-CMV-H2Bstaygold-SYN.

We generated pAAV2ss-CBA-H2B-mCherry-WPRE-bGH by removing the GFP gene from the plasmid and replacing it with the mcherry gene from pENTR4-H2B-mCherry and performing an LR clonase reaction (Gateway system, Invitrogen, Thermo Fisher Scientific, Zug, Switzerland) with pAAV2ss-CBA-RFA-WPRE-bGH. An H2B-staygold fragment (Geneart, Thermo Fisher Scientific, Zug, Switzerland) digested with AgeI/HindIII was used to replace the acGFPnuc gene from pENTR4-CMV-acGFPnuc to generate pENTR4-CMV-H2Bstaygold. Finally, the CMV-H2B-GFP fragment from pAAV2ss-gateway-CMV H2B-GFP-SYNpA was replaced with CMV-H2Bstaygold digested with NdeI/XhoI to generate pAAV2ss-gateway-CMV-H2Bstaygold-SYNpA.

Gene editing plasmids

The spCas9 in a SIN lentiviral backbone (SIN-cPPT-PGK-Cas9 (Church)-SRAD linker-SV40 nls-WPRE) has been described elsewhere ^[6](#_ENREF_6)^. The pAAV2ss-EFS-SpCas9-SYN plasmid was a gift from Ryohei Yasuda (Addgene; http://n2t.net/addgene:104588; RRID: Addgene_104588). For the editing of human ataxin-3, several guide RNAs were designed and initially ordered as single-stranded oligomers.

Exon 10 deletion strategy

An entry vector containing two U6-optimized tracrRNA cassettes (pMK-AttL1-U6-BsaI-tracrRNA-U6-SapI-tracrRNA-AttL2) was used to clone the sgRNAs for exon 10 deletion (sgATXN11 FWD: CACCGCATGAGAAAAACTATTCATA; REV: AAACTATGAATAGTTTTTCTCATGC; sgATXN12 FWD: ACCGACTTGGGAGTGATCTAGGTA; REV: AACTACCTAGATCACTCCCAAGTC; sgATXN13 FWD: ACCGAACATGATGAATGGTGAGC; REV: AACGCTCACCATTCATCATGTTC) and to generate the plasmids pMK-U6-sgATXN11, 12, 13, as well as pMK-U6-sgATXN11/12, pMK-U6-sgATXN11/13 (pMK-attl1-U6-sgATXN11-U6-sgATXN12-attl2, pMK-attl1-U6-sgATXN11-U6-sgATXN13-attl2). An LR reaction was then performed to transfer the expression cassettes to an AAV destination vector (pAAV2ss-RFA-bGH) and generate pAAV2ss-U6-sgATXN11-U6-sgATXN12-bGH and pAAV2ss-U6-sgATXN11-U6-sgATXN13-bGH.

Exon 9 strategy

An entry vector containing one U6-optimized tracrRNA cassette (pMK-AttL1-U6-BsaI-tracrRNA-AttL2) was used to clone the sgRNAs for exon 9 truncation (sgATXN14 FWD: CACCGATCTTACTTCAGAAGAGCTT; REV: AAACAAGCTCTTCTGAAGTAAGATC; sgATXN15 FWD: CACCGCAAGATATGACACAGACATC; REV: AAACGATGTCTGTGTCATATCTTGC; sgATXN16 FWD: CACCGCATATCTTGAGATATGTTTC; REV: AAACGAAACATATCTCAAGATATGC) and to generate the corresponding entry vectors (pMK-attL1-U6-sgATXN14-AttL2, pMK-attL1-U6-sgATXN15-AttL2, pMK-attL1-U6-sgATXN16-AttL2). An LR reaction was then performed to transfer the sgATXN14 (pMK-attL1-U6-sgATXN14-AttL2) to an AAV destination vector (pAAV2ss-RFA-bGH) and to generate pAAV2-U6-sgATXN14-SYNpolyA.

Ablate and replace strategy

The sgATXN1 and sgATNX2 guide RNAs target a region close to the translation start site of the human *ATXN3* gene (sgATXN1: FWD: CACCGCGGACACTCACTTTCTCG; REV: AAACCGAGAAAGTGAGTGTCCGC; sgATXN2: FWD: CACCGACTCACTTTCTCGTGGAAGA; REV: AAACTCTTCCACGAGAAAGTGAGTC). They were inserted into the pMK-attL1-U6-BsaI-tracrRNA-attL2 plasmid containing an optimized tracrRNA ^[6](#_ENREF_6), [7](#_ENREF_7)^ to generate pMK-attL1-U6-sgATXN1-tracrRNA-attL2 and pMK-attL1-U6-sgATXN2-tracrRNA-attL2, respectively. The lentiviral and AAV vectors were produced by transferring the sgRNA expression cassettes from the entry plasmids to LV and AAV destination vectors ^[3](#_ENREF_3)^ (pAAV2ss-U6-sgATXN1-bGH, pAAV2ss-U6-sgATXN2-bGH, pAAV2ss-U6-sgATXN2 CBA-H2B-staygold-SYNpA, SIN-cPPT-U6-sgATXN2-PGK-cherry-WPRE).

For identification of the transduced cells *in vivo*, a CMV-H2Bstaygold or CBA-H2Bstaygold cassette was added to AAV plasmids expressing the U6-sgRNA. For this, we generated new destination vectors (pAAV2ss-gateway-CMV H2Bstaygold-SYNpA; pAAV2ss-gateway-CBA H2Bstaygold-SYNpA (Geneart, Thermo Fisher Scientific, Zug, Switzerland)) for the transfer of all the sgRNAs.

A plasmid encoding the human ATXN3L paralog (pCCL-cPPT-CMV-human ATXN3L-Myc-DDK-WPREm, Origene, Herford, Germany) was used to demonstrate the specificity of the ATXN3L antibody used in the Jess capillary immunoassay (Figure 4).Optimization of the KamiCas9 system

The pMK-AttL1-NotI/BamHI-U6-BsaI-tracrRNAopt-BamHI/NcoI-U6-SapI-tracrRNA opt-NotI/NcoI/XbaI-7sk-BsmBI-tracrRNA-XbaI-AttL2 plasmid (#190898; Addgene) was used as the starting plasmid. The second U6 cassette and the 7sk promoter were deleted by BamHI/BsmBI digestion and replaced with the H1 promoter to generate pMK-Attl1-U6-SapI-tracrRNAopt-H1-BsmBI-tracrRNAorig-Attl2.

We generated a version with an H1 optimized tracr using the fragment (H1-tracrOPT: GCATCTAGAGAACGCTGACGTCATCAACCCGCTCCAAGGAATCGCGGGCCCAGTGTCACTAGGCGGGAACACCCAGCGCGCGTGCGCCCTGGCAGGAAGATGGCTGTGAGGGACAGGGGAGTGGCGCCCTGCAATATTTGCATGTCGCTATGTGTTCTGGGAAATCACCATAAACGTGAAATGTCTTTGGATTTGGGAATCTTATAAGTTCTGTATGAGACCACAGATCCCCGGAGACGGGTACCTCCGTCTCCGTTTCAGAGCTATGCTGGAAACAGCATAGCAAGTTGAAATAAGGCTAGTCCGTTATCAACTTGAAAAAGTGGCACCGAGTCGGTGCTTTTTTTGGTACCTCTAGATGC) to replace the 7sk-Orig cassette and to generate pMK-Attl1-U6-SapI-tracrRNAopt-H1-BsmBI-tracrRNAopt-Attl2.

We generated a version with a 7sk optimized tracr, using the fragment (CTGGGTACCTCGGAGACGGTAGGTCCGTCTCCGTTTCAGAGCTATGCTGGAAACAGCATAGCAAGTTGAAATAAGGCTAGTCCGTTATCAACTTGAAAAAGTGGCACCGAGTCGGTGCTTTTTTTGGTACCTCTAGto generate pMK-Attl1-U6-SapI-tracrRNAopt-7sk-BsmBI-tracrRNAopt-Attl2. We then cloned sgATXN2 and sgCas9_2 with the corresponding primers to generate the final entry plasmids. An LR reaction was performed with the AAV destination vector (pAAV2ss-Gateway-bGH) ^[3](#_ENREF_3)^ to generate pAAV2ss-U6-sgATNX2-H1-sgNish1-Orig-bGH, pAAV2ss-U6-sgATXN2-H1-sgNish1-OPT-bGH, pAAV2ss-U6-sgATXN2-7sk-sgNish-Orig-bGH, and pAAV2ss-U6-sgATXN2-7sk-sgNish1-OPT-bGH. The same expression cassettes were also inserted into an AAV destination vector expressing the H2B-staygold under the control of the chicken β-actin (CBA) promoter to produce pAAV2ss-U6-sgATNX2-H1-sgNish1-OPT-CBA-H2B-staygold-SYN, pAAV2ss-U6-sgATNX2-H1-sgNish1-Orig-CBA-H2B-staygold-SYN, pAAV2ss-U6-sgATNX2-7sK-sgNish1-OPT-CBA-H2B-staygold-SYN, and pAAV2ss-U6-sgATNX2-7sK-sgNish1-Orig-CBA-H2B-staygold-SYN.

Human wild-type ataxin-3 plasmid (CRISPR-resistant)

SIN-PGK-Myc-Ataxin3-27Q-WHV, encoding the wild-type human ataxin-3 cDNA in a SIN lentiviral backbone (SIN-cPPT-PGK-WPRE), has been described elsewhere ^[8](#_ENREF_8)^. The entry vectors pENTR4-human myc-ataxin3-27Q and pENTR4-human myc-ataxin3-(3QK-69Q), and destination vector pAAV2ss-CBA-Gateway-WPRE-bGH ^[3](#_ENREF_3)^ were used to generate pAAV2ss-CBA-Ataxin3-27Q-WPRE-bGH pAAV2ss-CBA-Ataxin3-69Q-WPRE-bGH. These plasmids are not a substrate for sgATXN2, as the target sequence of sgATXN2 is located in exon1 but also in intron 1, which is not present in these plasmids.

**Stereotaxic injections**

For the characterization of AAV2/rh.10 transduction patterns (Figure 1F-I), heterozygous MJD84.2 mice (*n*=3) received bilateral injections of 1.0x10^10^ vg AAV2/rh.10-CBA-GFP-WPRE-bGH. The animals were killed by sodium pentobarbital injection two weeks post-injection and transcardially perfused with PBS followed by 4% paraformaldehyde (4% PFA) (Fluka, Sigma, Buchs, Switzerland).

For demonstration of the co-expression of the H2B-cherry and H2B-GFP reporter genes in the cerebellum, heterozygous MJD84.2 mice (*n*=3) received bilateral injections of 5x10^9^ vg AAV2/rh.10-CBA-H2B-GFP-WPRE-bGH and 5x10^9^ vg AAV2/rh.10-CBA-H2B-cherry-WPRE-bGH per injection site (Figure 1J-O).

For the assessment of exon 10 deletion in the *ATXN3* gene, MJD84 mice (CTR: *n*=2 mice; 3 punches/animal, Treated: *n*=5; 3 punches/animal) received bilateral injections (2 sites/hemisphere 2.25x10^10^ vg per site in total) of 2.5x10^9^ vg AAV2/rh.10-EFS-SpCas9-SynPolyA, 2.0x10^10^ vg AAV2/rh.10-U6-sgATXN11-U6-sgATXN13, and 5.0x10^8^ vg AAV2/rh.10-CBA-AcGFPnuc-WPRE-bGH (Figure 1P).

We assessed the editing efficiency of the exon 9 truncation strategy, through bilateral injections into heterozygous MJD84.2 mice with (per site): Cas9 only group: 2.5x10^9^ vg AAV2/rh.10-EFS-SpCas9-SynPolyA and 5.0x10^8^ vg AAV2/rh.10-CBA-AcGFPnuc-WPRE-bGH (*n*=5), Exon 9 group: 5x10^9^ vg AAV2/rh.10-EFS-SpCas9-SynPolyA and 5.0x10^8^ vg AAV2/rh.10-CBA-AcGFPnuc-WPRE-bGH, 7.5x10^9^ vg AAV2/rh.10-U6-sgATXN14 (*n*=5).

We assessed the editing efficiency of the ablate strategy by bilateral injections into heterozygous MJD84.2 mice of the following (at each site): sgATXN2 only group: 5x10^9^ vg AAV2/rh.10-U6-sgATXN2 and 5.0x10^8^ vg AAV2/rh.10-CBA-AcGFPnuc-WPRE-bGH (*n*=3), ablate group: 5x10^9^ vg AAV2/rh.10-EFS-SpCas9-SynPolyA, 5x10^9^ vg AAV2/rh.10-U6-sgATXN2 and 5.0x10^8^ vg AAV2/rh.10-CBA-AcGFPnuc-WPRE-bGH (*n*=5).

For validation of the replace vector, heterozygous MJD84.2 (*n*=4) and C57BL/6 mice (*n*=4) received bilateral injections of the following (at each site): 5.0x10^8^ vg AAV2/rh.10-CBA-H2B-GFP-WPRE-bGH and 1.0x10^8^ vg AAV2/rh.10-CBA-cDNA-Ataxin3-27Q-WPRE-bGH.

For the proof-of-principle studies for the KamiCas9 ablate and ablate/replace strategies in the cerebellum, heterozygous MJD84.2 mice received bilateral injections at a single site per hemisphere (coordinates: -5.75 ; ±1.6 ; -2.6). In the first experiment, the reporter gene used was AAV2/rh.10-CBA-cDNA-ATXN3-27Q-WPRE-bGH, whereas, for the second experiment, we used AAV2ss-CBA-H2B-staygold-WPRE-bGH, which is better for fluorescence-activated nuclear sorting (FANS).

Cas9 only (control) group: 2.5x10^9^ vg AAV2/rh.10-EFS-SpCas9-SynPolyA and 5.0x10^8^ vg AAV2/rh.10-CBA-H2B-GFP/staygold-WPRE-bGH (*n*=5).

Replace group: 2.5x10^9^ vg AAV2/rh.10-EFS-SpCas9-SynPolyA, 1.0x10^7^ vg AAV2/rh.10-CBA-cDNA-ATXN3-27Q-WPRE-bGH and 5.0x10^8^ vg AAV2/rh.10-CBA-H2B-GFP/staygold-WPRE-bGH (*n*=5),

Ablate group: 2.5x10^9^ vg AAV2/rh.10-EFS-SpCas9-SynPolyA, and 5.0x10^8^ vg AAV2/rh.10-CBA-H2B-GFP/staygold-WPRE-bGH and 7.5x10^9^ vg AAV2/rh.10-U6-sgATXN2 (*n*=5),

Ablate and replace group: 2.5x10^9^ vg AAV2/rh.10-EFS-SpCas9-SynPolyA, and 5.0x10^8^ vg AAV2/rh.10-CBA-H2B-GFP/staygold-WPRE-bGH and 7.5x10^9^ vg AAV2/rh.10-U6-sgATXN2 and 1.0x10^7^ vg AAV2/rh.10-CBA-cDNA-ATXN3-27Q-WPRE-bGH (*n*=5),

KamiCas9 ablate group: 2.5x10^9^ vg AAV2/rh.10-EFS-SpCas9-SynPolyA, and 5.0x10^8^ vg AAV2/rh.10-CBA-H2B-GFP/staygold-WPRE-bGH and 7.5x10^9^ vg AAV2/rh.10-U6-sgATXN2-7sksgCas9 (*n*=5),

KamiCas9 ablate and replace group: 2.5x10^9^ vg AAV2/rh.10-EFS-SpCas9-SynPolyA, and 5.0x10^8^ vg AAV2/rh.10-CBA-H2B-GFP/staygold-WPRE-bGH and 7.5x10^9^ vg AAV2/rh.10-U6-sgATXN2-7sksgCas9 and 1.0x10^7^ vg AAV2/rh.10-CBA-cDNA-ATXN3-27Q-WPRE-bGH (*n*=5). Mice were killed 8 weeks post-injection, for DNA extraction.

**Qiacuity digital PCR analysis**

The absolute number of ATXN3 copies was determined with 180 ng genomic DNA, the QIAcuity Probe PCR Kit (Qiagen, Basel, Switzerland) and 0.8 μM primers targeting intron 9 of the human *ATXN3* gene (FWD: GACCTGAGTGAAAAGAATGA, REV TTGATCAGATAAAGCATTTCAAAC and 0.4 μM of FAM-BHQ1 probe CCATGGAAACATTATGTTAACTCACAT)(Jackson Laboratory, Bar Harbor, USA). The ATXN3 amplification signal was normalized against the amount of gDNA for the poly(rC)-binding protein 2 (PCBP2) gene ^[9](#_ENREF_9)^, with the corresponding primers and probes (FWD TTGTGTCTCCAGTCTGCTTG, REV AGGTGGTGGTGGTGGTA and VIC-BHQ1 CCCTCTCCTGGCTCTAAATGTTGTGT) (Microsynth, Balgach, Switzerland). Loaded plates were subjected to EcoRI-HF digestion for 10 min, followed by PCR as follows: initial denaturation at 95 °C for 2 min, 40 cycles of 95 °C, 60 °C and 72 °C for 15 s each for amplication and a final step at 40 °C for 5 min.

**PCR and TIDE analysis on HEK293T cells and NPCs**

For sgRNA screening in HEK293T cells, the *ATNX-3* gene was amplified with the following sets of PCR primers.

Exon 10 deletion (sgATXN11, 12, 13): For the single-cut TIDE analysis of sgATXN12 and sgATXN13: We used the FWD: AGCACTTCCATATTTTAAAGTAATCTG and REV: TTTCCCTGGATTAAGGAGCA primers for PCR, with annealing at 56 °C, 40 cycles and a final elongation step of 20 s. For exon 10 deletion, the following primers were used: FWD: AGCACTTCCATATTTTAAAGTAATCTG, REV: TTTCCCTGGATTAAGGAGCA. PCR was performed with an annealing temperature of 56°C, for 35 cycles, with a final elongation step of 20 s.

Exon 9 truncation (sgATXN14, 15, 16): The PCR for TIDE analysis was performed with the following primers: FWD: TCTTGCCTCTATGCATGCCTCT; REV: TCACAGGATTCAGGCAGTAACC, at an annealing temperature of 59 °C, for 35 cycles, with a final elongation step of 20 s.

Ablate and replace (sgATXN1, 2): The PCR for TIDE analysis was performed with the following human *ATXN3* primers: FWD: GAGGACGCGCTACCAAGG, REV: GCAGGCTAGGCAGACTACAA, at an annealing temperature of 63 °C, for 35 cycles, with a final elongation step of 20 s.

The editing of the human ATXN3L was performed with the specific primers FWD: CCATGGTGTCCATTCGGTTAAA, REV: GGCTAATTCCACAGGGCTAAAA at an annealing temperature of 56 °C, for 40 cycles, with a final elongation step of 20 s.

The data were normalized against the H2B-staygold encoded by the plasmid expressing the sgRNAs, with 50 ng of gDNA. We used the FWD: TGAGCGACAAGAGCAAGTG, and REV primers: TTGCTCTGGGTGTACTGCTT for PCR at an annealing temperature of 60 °C, for 40 cycles, with a final elongation step of 20 s.

We used the following primers to quantify Cas9 editing: Cas9_2 FWD: TTTTTCGCAACGGGTTTGCC and Cas9_2 REV AGAAGCTGTCGTCCACCTTG. PCR was performed at an annealing temperature of 65 °C, for 40 cycles, with a final elongation step of 15 s.

**PCR and TIDE analysis on cerebellar punch specimens and isolated nuclei**

In the proof-of-principle study with sgATNX2 in MJD84.2 mice (*n*=3 Ctr and *n*=5 treated mice), editing efficiency was analyzed with human-specific *ATXN3* primers (FWD: GAGGACGCGCTACCAAGG and REV: GCAGGCTAGGCAGACTACAA). PCR was performed with the GAAAGTGACGGAGAAAGG primer at an annealing temperature of 59 °C, for 32 cycles, with a final elongation step of 20 s. We also analyzed the editing of the mouse *Atxn3* gene with mouse-specific primers (FWD: CCTAAAACTTGGCTGCGAGGA and REV: CCGAAGTGTCAGGCCCTTTA). PCR was performed with the TAGAGAAGTTCCTCCGCACAGC primer at an annealing temperature of 61 °C, for 32 cycles, with a final elongation step of 20 s. Punch specimens from Ctr (*n*=12) and treated mice (*n*=13 for human *ATX3*, *n*=20 for mouse *Atxn3*) were analyzed. Cas9 editing was performed as described for HEK293T cells.

**REFERENCES**

1. Zala D, Benchoua A, Brouillet E, Perrin V, Gaillard MC, Zurn AD *et al.* Progressive and selective striatal degeneration in primary neuronal cultures using lentiviral vector coding for a mutant huntingtin fragment. *Neurobiol Dis* 2005; **20**(3)**:** 785-98.

2. Kanda T, Sullivan KF, Wahl GM. Histone-GFP fusion protein enables sensitive analysis of chromosome dynamics in living mammalian cells. *Curr Biol* 1998; **8**(7)**:** 377-85.

3. Duarte F, Ramosaj M, Hasanovic E, Regio S, Sipion M, Rey M *et al.* Semi-automated workflows to quantify AAV transduction in various brain areas and predict gene editing outcome for neurological disorders. *Molecular therapy. Methods & clinical development* 2023; **29:** 254-270.

4. Hirano M, Ando R, Shimozono S, Sugiyama M, Takeda N, Kurokawa H *et al.* A highly photostable and bright green fluorescent protein. *Nat Biotechnol* 2022; **40**(7)**:** 1132-1142.

5. Nishiyama J, Mikuni T, Yasuda R. Virus-Mediated Genome Editing via Homology-Directed Repair in Mitotic and Postmitotic Cells in Mammalian Brain. *Neuron* 2017; **96**(4)**:** 755-768 e5.

6. Merienne N, Vachey G, de Longprez L, Meunier C, Zimmer V, Perriard G *et al.* The Self-Inactivating KamiCas9 System for the Editing of CNS Disease Genes. *Cell Rep* 2017; **20**(12)**:** 2980-2991.

7. Dang Y, Jia G, Choi J, Ma H, Anaya E, Ye C *et al.* Optimizing sgRNA structure to improve CRISPR-Cas9 knockout efficiency. *Genome Biol* 2015; **16:** 280.

8. Alves S, Regulier E, Nascimento-Ferreira I, Hassig R, Dufour N, Koeppen A *et al.* Striatal and nigral pathology in a lentiviral rat model of Machado-Joseph disease. *Hum Mol Genet* 2008; **17**(14)**:** 2071-83.

9. Christodoulou I, Patsali P, Stephanou C, Antoniou M, Kleanthous M, Lederer CW. Measurement of lentiviral vector titre and copy number by cross-species duplex quantitative PCR. *Gene Ther* 2016; **23**(1)**:** 113-8.
